# Anxiety-depressive disorders among irritable bowel syndrome patients in Guilan, Iran

**DOI:** 10.1186/1756-0500-5-112

**Published:** 2012-02-21

**Authors:** Mohamad-Jafar Modabbernia, Fariborz Mansour-Ghanaei, Ali Imani, Seyed-Alireza Mirsafa-Moghaddam, Massih Sedigh-Rahimabadi, Mahmoud Yousefi-Mashhour, Farahnaz Joukar, Zahra Atrkar-Roushan, Siamak Bidel

**Affiliations:** 1 Department of Psychiatry, Guilan University of Medical Sciences, Rasht, Iran; 2 Gastrointestinal and Liver Diseases Research Center, Guilan University of Medical Sciences, Rasht, Iran; 3 Hijelt Institute, University of Helsinki, Helsinki, Finland; 4 National Institute for Health and Welfare, Helsinki, Finland

**Keywords:** IBS, Psychiatric disorders, Depression, Anxiety

## Abstract

**Background:**

Psychiatric disorders are common in irritable bowel syndrome (IBS) patients. The prevalence of psychiatric disorders in IBS patients varies in different cultures. We conducted this study to determine the prevalence of psychiatric disorders

**Methods:**

In a cross-sectional study, 256 IBS patients were selected (using the criteria of Rome III) and evaluated for psychiatric disorders. In the first phase, subjects were screened using the General Health Questionnaire 28 (GHQ28). In the second phase, those who had scores ≥ 23 were assessed through semi-structured psychiatric interviews.

**Results:**

Thirty out of 256 subjects had no significant psychiatric symptoms after performing GHQ28. In further psychiatric evaluation of the remaining subjects (226) who suffered from some degree of a psychiatric problem, 36 were diagnosed without Anxiety/Depressive disorder. Thus 66 subjects (25.8%) were known as a group without any significant psychiatric problem. A total of 190 subjects (74.2%) with anxiety-depressive problems were diagnosed; 89 were suffering from pure anxiety disorders, 41 were suffering from depressive disorders and 60 had co-morbid anxiety-depressive disorders. When comparing anxiety-depressive patients (n = 190) with normal subjects (n = 66), gender (P = 0.016), occupation (P = 0.002) and intensity of IBS (P < 0.001) showed statistically significant differences.

**Conclusion:**

The high prevalence of anxiety-depressive disorders in this study indicates the necessity of psychiatric assessment, early diagnosis and treatment of the patients with IBS. It may improve management of the patients suffering from IBS.

## Background

Irritable bowel syndrome (IBS) is a malfunction of the intestine that is characterized by abdominal pain and/or abdominal discomfort and changes in bowel habits without any detected structural disorders. Ten to twenty percent of adults and teenagers worldwide have some signs of IBS. It is more common in females and less common in the elderly (more than 60 years old). Less than one-third of these patients visit practitioners, which accounts for about 12% of primary care visits. The severity of symptoms is varied and it may have some effects on patient quality of life and increases healthcare costs [1]. The pathophysiology of IBS is still poorly understood, thus the treatment of IBS is mainly directed toward symptoms.

It has been shown that patients with the most intense symptoms have the worst quality of life, and the morbidity of IBS is equal to severe organic gastrointestinal diseases. IBS is the second cause of absence from work (after the common cold) and it is almost 3 times more than other workers [2]. In a survey of 8 European countries, a randomized cluster sampling of 41,984 individuals, patients with IBS were identified. After psychiatric evaluation, 78 percent of them believed that IBS had some effect on their health state and quality of life which in 24% these effects estimated as very significant [3].

Several studies have evaluated the relation between IBS and psychiatric disorders [4-6]. It has been reported that neurosis, anxiety, depression, and dysfunctional cognition are more prevalent in patients with IBS [7-11]. In a large randomized controlled trial Guthrie et al. found that 44% of IBS patients had psychiatric comorbidity which depressive and anxiety disorders were the most common conditions [12]. In a study by Yates, six major gastrointestinal features including: abdominal pain, diarrhea, bloating, constipation, loss of appetite and vomiting were considered along with the psychiatric state of the patients. Major depression (13.4%), panic disorder (12.5%) and agoraphobia (17.8%) were found to be more common in patients with 2 or more GI symptoms [13]. A most recent study provided further evidence that GI specific anxiety is an important mediating factor affecting the GI symptom severity and quality of life in IBS patients. This study concluded that reduction of gastrointestinal specific anxiety in IBS could be a relevant treatment goal to achieve general improvement in patients with IBS [14]. The prevalence of anxiety-depressive disorders in IBS patients differs according to the studied population, study type, sample size and methodology of the research. In northern part of Iran, there was no available data on the psychiatric evaluation of the patients with IBS. Therefore this study was designed to determine the prevalence of psychiatric disorders in the IBS patients of the Guilan province.

## Methods

In this cross-sectional study we included patients diagnosed as IBS cases. In a preliminary pilot study on 24 IBS patients, the prevalence of phobia, major depression, generalized anxiety and dysthymia were 29.4%, 29.4%, 41.2% and 41.2%, respectively. Therefore, considering α = 0.05, d = 0.056 and prevalence = 29.4%, (N = P.(1-P).Z^2^/d^2^), we enrolled 256 IBS patients in this study. These patients were recruited from gastroenterology private clinics, and then were referred to the "Psychiatrics" who worked in Gastrointestinal and Liver Disease Research Center (GLDRC), Rasht the capital city of Guilan province in northern Iran. The gastroenterologists chose them randomly from IBS patients who had been referred to their private clinics during January 2009 to June 2009. They referred the first one then omitted the second and referred the third one and so on. Therefore, 256 subjects out of 512 IBS patients were included in this study. The inclusion criteria were: diagnosed with IBS by physician according to the Roma III criteria, age between 15-80 and willing to enroll in the research.

This study was performed in two phases. In the first phase, the probable cases of depression or anxiety were detected with the self-reporting General Health Questionnaire 28 (GHQ28). In the second phase, the patients who scored > 23 of GHQ28, underwent a semi-structured clinical interview using the DSM IV-TR to evaluate anxiety-depressive disorders.

The standardized GHQ28 test in Iran was shown to have a sensitivity and specificity of 84 - 88% and 78 - 93%, respectively, validity of 85 - 90%, an overall classified error of 8.2 - 19%, best scoring of 0, 1, 2 and 3 and best cut off point of 23 [15]. The questionnaire had four scales (somatic, anxiety and sleeping disorders, major depression, and social dysfunction). Each scale consisted of 7 questions and each question had four choices with a score of 0 to 3. Two residents of psychiatry, who were qualified on using the semi-structural Diagnostic and Statistical Manual of Mental Disorders, 4^th ^edition (DSM IV-TR) interview and after reaching an acceptable correlation rate, initiated the survey. The IBS cases that had previously been diagnosed using the Rome III criteria [16] were randomly selected from the gastroenterology private clinics. Demographic information (e.g., age, sex, occupational and marital status and educational level) was obtained and the GHQ28 was given to them to be filled out in 20 min. In the case of illiterate participants, the interviewer assisted them with filling out the forms.

From 256 patients, who referred by the gastroenterologists, 30 had a GHQ 28 < 23 so they considered normal and from the remaining (226); 36 were without Anxiety/Depressive disorder, although, they had some psychiatric signs, they did not have any psychiatric disorders. Thus we had 190 IBS patients with Anxiety-Depressive disorders compare with 66 IBS patients without any psychiatric disorders.

### Psychiatric assessment

Judgment on the psychiatric state of studied IBS patients was carried out using the GHQ28 and semi-structured psychiatric interviews and patients were subsequently divided into 4 groups as presented in Table 1.

**Table 1:** Psychiatric state assessment of the IBS patients [17]

Normal	Patients with a GHQ28 score < 23.
**Depression**	The presence of at least one of the following disorders: major depressive disorder (MDD), minor depressive disorder or dysthymic disorder.

**Anxiety**	The presence of at least one of the following disorders: phobia, panic attacks, generalized anxiety disorder (GAD), obsessive-compulsive disorder (OCD) or post-traumatic stress disorder (PTSD).

**Co-morbid**	Having both anxiety and depression criteria simultaneously.

Diagnostic criteria for IBS [11] were defined as having recurrent abdominal pain or discomfort at least 3 days per month in the last 3 months associated with 2 or more of the following:

1. Improvement with defecation

2. Onset associated with a change in frequency of stools

3. Onset associated with a change in form (appearance) of stools

These criteria were fulfilled for the previous 3 months with symptom onset at least 6 months prior to diagnosis.

### Subtyping IBS by predominant stool pattern

Subtyping IBS using the predominant stool pattern [16] was carried out using the criteria presented in Table 2.

**Table 2:** Subtyping IBS using the predominant stool pattern [17]

IBS with constipation (IBS-C)	Hard or lumpy stools for ≥ 25% of bowel movements and loose (mushy) or watery stools for < 25% of bowel movements.
IBS with diarrhea (IBS-D)	Loose (mushy) or watery stools for ≥ 25% of bowel movements and hard or lumpy stool for < 25% of bowel movements.

Mixed IBS (IBS-M)	Hard or lumpy stools for ≥ 25% of bowel movements and loose (mushy) or watery stools for ≥ 25% of bowel movements.

Unsubtyped IBS	Insufficient abnormality of stool consistency to meet criteria for IBSC, IBS-D or IBS-M.

Although the above sub-classifications may be used to subtype patients according to bowel habits, the validity and stability of such subtypes over time is unknown. IBS severity scores were based on five visual analogue scales: severity of pain, duration of pain, abdominal bloating, bowel satisfaction and quality of life. Overall IBS severity scores were expressed as a score from 0 (least severe) to 500 (most severe). Mild, moderate and severe cases were indicated by scores of 75 to 175, 175 to 300 and > 300, respectively [18]. The participants were asked about the occupational and educational status as well.

### Statistics analysis

We used descriptive tools for prevalence analysis, chi-square tests for comparing qualitative variables (e.g., IBS and depression), T-tests for quantitative variables and kappa tests (> .75) for detecting the agreement coefficient between interviewers. P-values < 0.05 were considered statistically significant.

### Ethical considerations

This study was approved by the ethic committees of Guilan Gastrointestinal and Liver Disease Research Center (GLDRC). We fully explained the study to the participants (IBS patient with or without anxiety/depressive disorder) and written consents were obtained. All private information remained confidential and, when needed, patients were referred to a psychiatrist for appropriate treatment.

## Results

**Figure 1:**
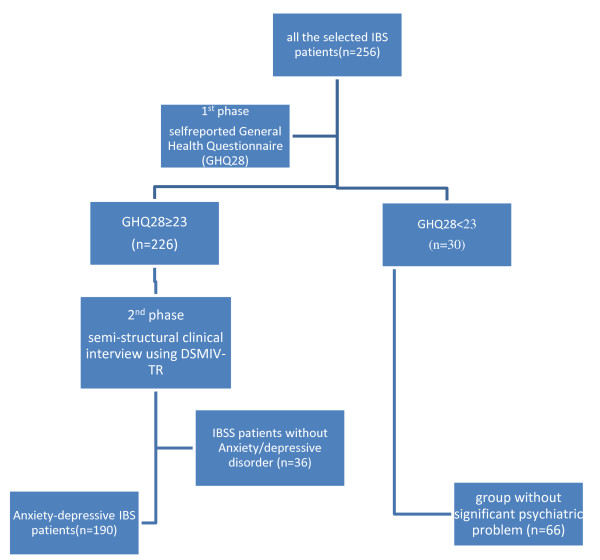
**Study action plan**.

**Figure 2:**
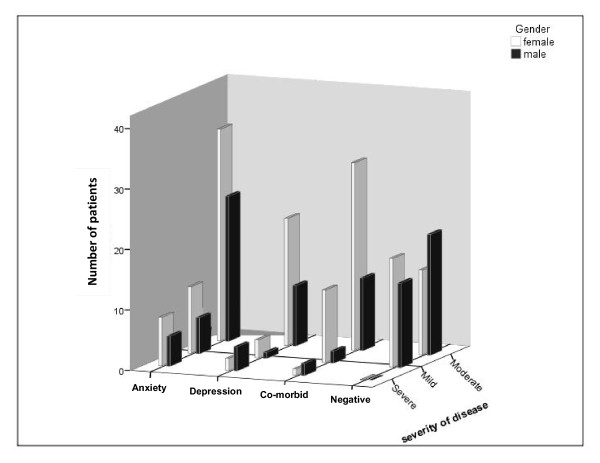
**Distribution of psychiatric disorders and severity of IBS between females and males**.

Of 256 patients participated in this study, 156 (60.9%) were females and 100 (39.1%) were males and 226 subjects (83 males and 143 females) suffered from a degree of psychiatric problems (GHQ28 ≥ 23). After performing semi-structured psychiatric interviews, 190 subjects (74.2%) had anxiety-depressive disorders (89 were suffering from anxiety disorders, 41 depressive disorder and 60 had co-morbid anxiety-depressive disorders; Figures 1, 2).

The demographic variables of gender (P = 0.016), occupation (P = 0.002) and intensity of IBS (P < 0.001), in comparison with other subjects (66), showed statistically significant differences (Table 3).

**Table 3:** Different variables among IBS patient with or without anxiety/depressive disorder

Variable	Group	Presence of Anxiety/Depressive isorder(256)		*P*-value*
			
		Negative(66)	Positive(190)	
Gender	Male	34 (34%)	66 (66%)	**0.016**
		
	Female	32 (20.5%)	124 (79.5%)	

Marital Status	Single	16 (34.8%)	30 (65.2%)	**0.445**
		
	Married	49 (23.9%)	156 (76.1%)	
		
	Widow or	1 (20%)	4 (80%)	
		
	Divorce			

Occupational Status	Jobless	27 (18.4%)	120 (81.6%)	**0.002**
		
	Has a job	39 (35.8%)	70 (64.2%)	

Type of IBS	Diarrhea	24 (21.4%)	88 (78.6%)	**0.11**
		
	Constipation	34 (32.7%)	70 (67.3%)	
		
	Mixed	8 (20%)	32 (80%)	

Severity of disease	Mild	32 (47.8%)	35 (52.2%)	**0.001**
		
	Moderate	34 (20.4%)	133 (79.6%)	
		
	Severe	0	22 (100%)	

Educational Status	Under diploma	25 (21%)	94 (79%)	**0.104**
		
	Diploma or higher	41 (29.9%)	96 (70.1%)	

Age group	< 21	3 (25%)	9 (75%)	**0.971**
		
	21-39	39 (26.4%)	109 (73.6%)	
		
	≥ 40	24 (25%)	72 (75%)	

*P-values > 0.05 are considered non-significant (NS)

In anxiety disorder cases, GAD was the most frequent, and in depressive disorder cases, dysthymia was the most frequent. There were statistically significant differences in age [mean age of 42.7 (s.d. = 13.4) vs. 37.1 (s.d. = 12.4), P = 0.033], severity of disease (P < 0.001), educational level (P = 0.016) and marital status (P = 0.014) between patients with depressive disorders (n = 41) compared with psychiatrically normal cases (n = 66). Comparing IBS patients that had anxiety disorders and psychiatrically normal cases, there were significant differences in the severity of disease (P < 0.001) and occupational status (P = 0.002). Also, comparing patients with anxiety and those with depressive disorders, IBS patients with anxiety were younger [mean age of 33.0 (s.d. = 10.6) vs. 42.7 (s.d. = 13.4) and P = 0. 01], more frequently single (P = 0.01), had a higher educational status (P = 0.024) and suffered more from IBS-D (P = 0.01; Table 4).

**Table 4:** Distribution of different variables among IBS patient with anxiety-depressive disorders

Variable	Group	Anxiety-depressive disorders(190)			*P*-value*	*P*-value**
				
		Anxiety(89)	Depression(41)	Co- morbid(60)		
Gender	Male	35(53.1%)	15 (22.7%)	16 (24.2%)	0.765***	0.271
			
	Female	54(43.5%)	26 (21%)	44 (35.5%)		

Marital Status	Single	19(63.3%)	3 (10%)	8 (26.7%)	< 0.01	0.104
			
	Married	70(44.9%)	36 (23.1%)	50 (32%)		
			
	Widow or divorce	0	2 (50%)	2 (50%)		

Occupational Status	Jobless	56(46.7%)	23 (19.2%)	41 (34.2%)	0.459	0.456
			
	Has a job	33(47.1%)	18 (25.7%)	19 (27.2%)		

Type of IBS	Diarrhea	50(56.8%)	12 (13.7%)	26 (29.5%)	< 0.01	0.02
			
	Constipation	25(35.7%)	18 (35.7%)	27 (38.5%)		
			
	Mixed	14(43.7%)	11 (34.4%)	7 (21.9%)		

Severity of disease	Mild	17(48.5%)	4 (11.4%)	14 (40.1%)	0.39	0.189
			
	Moderate	59(44.3%)	31 (23.3%)	43 (32.3%)		
			
	Severe	13(59.1%)	6 (27.3%)	3 (13.7%)		

Educational Status	Under diploma	37(39.4%)	26 (27.7%)	31 (33%)	0.02	< 0.05
			
	Diploma or higher	52(54.2%)	15 (15.6%)	29 (30.3%)		

Age group	< 21	6 (64.7%)	0	3 (33.3%)	< 0.01	< 0.01
			
	21-39	62(56.9%)	16 (14.6%)	31 (28.5%)		
			
	≥ 40	21(29.3%)	25 (34.8%)	26 (36%)		

*P-values represent differences between anxiety and depression

**P-values represent differences in anxiety-depressive disorders

***P-values > 0.05 are considered non-significant (NS)

## Discussion

Several authors have emphasized the need for psychological assessment in patients with functional bowel disorders [10,19-21]. Different studies have shown that almost two-thirds of all patients with self-reported depressive symptoms were not identified by the attending physician as suffering from psychiatric problems [22,23]. Similarly, physicians were shown to detect only one-third of all cases in somatoform disorders [24]. Therefore, psychological assessment should be mandatory in patients with functional bowel disorders. The screening of patients for anxiety and depression can be performed easily through patient questionnaires and is the basis and justification for additional therapeutic interventions, which have been shown to be effective in the GI symptom severity and quality of life of the IBS patients [14,25-27]. At the present time, the treatment of IBS patients in clinical practices focuses on physical symptom relief. The efficacy of various medical and dietary treatments has not been established [21,28] in contrast to the efficacy of various psychological interventions. Recently, Zijdenbos et al. [27] suggested that psychological interventions are superior to "care as usual" for improvement of symptoms. In a systematic review and meta- analysis Ford et al. demonstrated a significant benefit of antidepressants over the placebo, and psychological therapies over control therapy or a physician's "usual management", for the treatment of IBS [25].

In our study, the prevalence of psychiatric disorders in IBS patients was evaluated in Guilan, a northern province of Iran. Among 256 IBS patients that participated in this survey, 190 (74.2%) had one of the anxiety-depressive disorders. Approximately 3 of 5 IBS patients in our study had at least one kind of anxiety disorder and 2 showed some degree of depression. The main findings of our study were the importance of psychiatric disorders, and especially anxiety, in the severity of IBS. Most IBS patients with anxiety were married, had a higher level of education, were unemployed, suffered from IBS-D and had severe symptoms. The high prevalence of anxiety and depression in the local general population (20.8% and 21%, respectively) [15] must be considered as an important factor in the higher prevalence of these diseases in our studied IBS patient group (35.5% and 16%, respectively). Studies with an adequate number of patients and a standardized psychiatric interview indicated that 50% to 60% of IBS patients in gastroenterology clinics have psychiatric disorders. The proportion is similar in patients entering treatment trials for IBS symptoms [29]. North American studies have found that anxiety disorders are highly prevalent in IBS outpatients (39.7-52.4%) [30]. However, we cannot exclude those patients recruited via advertisement and have consulted a physician due to their IBS symptoms previously. In this study, the prevalence of anxiety-depressive disorders in IBS patients was higher in females, which is similar to Blanchard et al. study [31,32]. This difference may explain by the higher prevalence of anxiety and depression in the female normal population [17,33,34]. Also, the prevalence of IBS in general population has been reported higher in women [35]. However, a previously published paper, which examined gender differences among IBS patients in degree of psychological distress, concluded that it might be a function of the method of measurement. This means that by employing some validated tests, such as the Beck Depression Inventory (BDI), State-Trait Anxiety Inventory (STAI), or Minnesota Multiphasic Personality Inventory (MMPI), female IBS patients showed more psychological distress. However, by using the categorical approach of the DSMs, it seems that male and female IBS patients do not differ [30].

In our study the presence of possible depression, as well as a higher mean level of depression symptoms, was more frequent in females than males, but was not statistically significant. This was similar to Cain et al. study, [36] in which no sex differences were found with regard to depression symptoms in IBS patients. Possible anxiety disorders showed a trend to be more prevalent in female patients in our study. Also, the mean level of anxiety symptoms was not different between males and females. The most frequent type of anxiety disorder in our study was GAD, which is consistent with previously reported data [31,37]. In our analysis, comparing IBS patients with and without psychiatric disorders, a significant association with the severity of the disease has been observed (P < 0.001). This might be considered as clear evidence that all IBS patients who suffer from a severe type of disease could have some degrees of anxiety-depressive disorder.

Recent published results by Thijssen et al. showed the importance of anxiety and depression on quality of life and symptom severity in IBS patients. IBS patients with anxiety disorder or depression had more severe symptoms, a worse physical and mental quality of life score, and higher levels of dysfunctional cognition [38]. It has also been suggested that anxiety about gastrointestinal symptoms, not general anxiety or depression, may be more relevant for symptom severity and health outcomes in IBS and other functional GI disorders [39].

Comparing IBS patients with depression (41 cases) to IBS patient without anxiety-depressive disorders (66 cases), depressed patients were older (P = 0.033), more frequently married (P = 0.014), suffered more from a severe type of IBS (P < 0.001) and had a lower educational level (P = 0.016). It seems that there is a high prevalence of depressive disorder in our society [15] and the relation between depression and age, educational level, marital and job status have some confounding effects on observed results in our study.

Psychosocial stress and psychopathology are increased in functional disorders, but not in all individuals, and is generally thought of as being a cause rather than the consequence of functional disorders [40]. This study may provide indicative evidence for IBS as a disorder with a psychosomatic aspect.

Although research methodology, study population, measuring tools, sampling and sample sizes are some factors that may influence the outputs of surveys, we may suggest that the severity of IBS has a significant relation with the presence of anxiety-depressive disorders. Patients who suffer from possible anxiety disorders or depression had more severe symptoms. Suffering from a possible anxiety disorder independently influences IBS patients and may have a significant impact on symptom severity. The distribution of these psychiatric problems among our patients had some relation with their age, educational level and type of IBS.

A possible limitation of this study is the recruitment of patient data from a two-step examination. However, this is unlikely to have any effects on the outcome. We believe that GHQ28is a legitimate test, however in patients with scores < 23 performing DSM IV-TR would be more effective. Different commodity variables could increase the prevalence of mood disorders but these results should be interpreted with a great caution and would need more researches in the future.

## Conclusion

There was a high prevalence of anxiety-depressive disorders in our subjects. Age, educational level and type and severity of IBS had remarkable distributional differences among the studied groups. Also, psychological factors influence symptom severity in IBS patients. This data emphasize the importance of the psychological evaluation of the patients with IBS to identify those who might be suffering from psychological disorders in order to better management of the patients and may also help to reduce the burden of health care costs.

Further studies will be needed to assess the outcome of the psychiatric evaluation and treatment in symptom relief of the IBS patients.

## Competing interests

The authors declare that they have no competing interests.

## Authors' contributions

MJM, FMG, AI, SAMM, SB, have made substantial contributions to conception and design, acquisition of data, analysis and interpretation of data. MJM, FMG, AI, SAMM, MSR, MYM, FJ, ZAR, SB, have been involved in drafting the manuscript or revising it critically for important intellectual content. All authors read and approved the final version of the paper.
